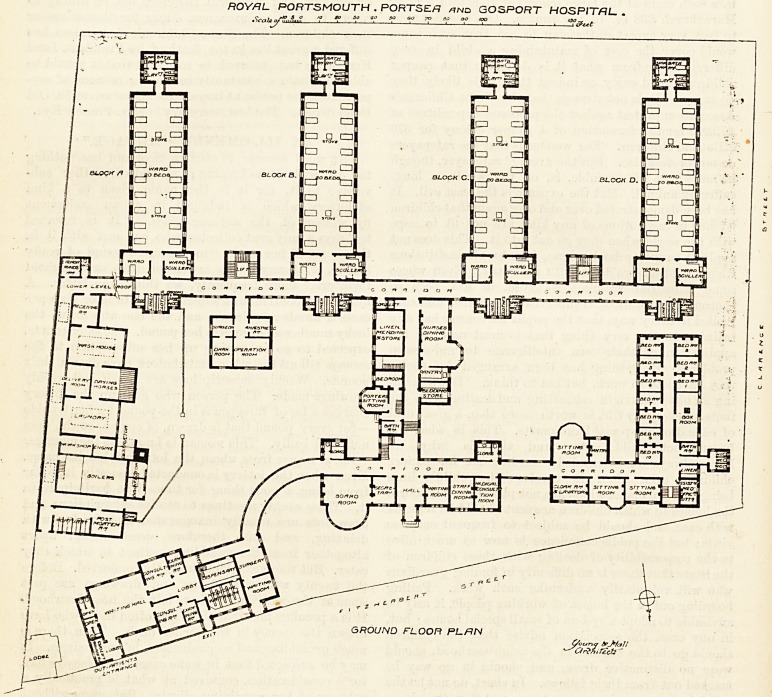# Hospital Construction

**Published:** 1899-07-01

**Authors:** 


### The Institutional Workshop.

### HOSPITAL CONSTRUCTION.

### THE NEW ROYAL PORTSMOUTH HOSPITAL.

The foundation of the Royal Portsmouth, or, to give its full title, the Royal Portsmouth, Portsea, and Gosport Hospital, dates from 1846, in which year a site was granted to the town by the Board of Ordnance. The foundation-stone of the new building was laid on September 27th, 1847, by H.R.H. Prince Albert, and the building was completed and ready fo occupation on January 2nd, 1849. The number of beds provided was 20; by subsequent additions this has been increased to 136. The old buildings are, as may be imagined, anything but suitable for their purpose, neither do they provide sufficient accommodation for the needs of the town. Acting on the advice of their architects the committee wisely determined not to adopt a policy of tinkering or adding to the old buildings, but to lay out a complete scheme for a new hospital, and to proceed with the work piecemeal as opportunity and funds may permit. The plan we publish to-day shows the complete scheme of rebuilding. The parts just finished are two pavilions, with their staircase blocks. The erection of these two blocks has not interfered in any way with the working of the hospital, nor has it necessitated the removal of any part of the existing buildings. The pavilions are two storeys in height and are raised 5 ft. above the ground level, a clear air space being formed under each lower storey, freely ventilated on both sides. Each pavilion contains a large ward for twenty beds, with a small one-bed ward for special cases, a ward kitchen, bath-room, and the necessary sanitary offices, and cupboards for linen, food, and patients' clothes. The wards are warmed by means of double Teale grates, two pairs in each ward, with descending flues, and ventilation is provided for by windows. The floors are of terrazzo, and the walls and ceilings are painted and varnished. The plans have been designed and the erection of the buildings supervised by Messrs. Keith Young and Hall.

## Figures and Tables

**Figure f1:**